# Effect of Ice Consistency and Sodium Chloride Additives on Cooling Speed and Final Temperature for Cold Water–Ice Immersion in Heat Stroke

**DOI:** 10.5811/westjem.48490

**Published:** 2026-02-22

**Authors:** Andrew Goldmann, Bryan Yavari, David Sklar

**Affiliations:** * Creighton University School of Medicine, Emergency Medicine Residency, Phoenix, Arizona; † Arizona State University, College of Health Solutions, Phoenix, Arizona; ‡ University of Arizona College of Medicine–Phoenix, Phoenix, Arizona

## Abstract

**Introduction:**

Heat stroke can rapidly progress to end organ damage and death if not promptly treated. The diagnosis is characterized by core body temperature > 40.5 °C. In this study we evaluate how the form of ice (crushed vs cubed), the addition of sodium chloride, and the initial temperature of water together affect the rate of cooling for standardized cooling bath mixtures used to treat patients experiencing heat stroke.

**Methods:**

We prepared four cold water immersion mixtures using 12 quarts of ice and 12 quarts of water (11.36 liters) under different conditions:

Test Case 1: Cubed ice with trauma bay tap water (~35 °C);
Test Case 2: Crushed ice with cold tap water (~24 °C);
Test Case 3: Crushed ice with cold tap water plus four pounds of rock salt;
Test Case 4: Cubed ice with cold tap water,

After each mixture was poured into a 40-quart bucket and mixed thoroughly, we recorded the temperature at 20-second intervals over a total duration of 300 seconds using a food-grade thermometer. Room temperature during the experiment was 25.0 °C.

**Results:**

After 100 seconds, water from the trauma bay with cubed ice reached 6.2 °C, while cold tap water with cubed ice cooled to a slightly lower temperature of 5.5 °C. Crushed ice in cold tap water reached an even lower temperature of 3.6 °C. The coldest mixture was made with crushed ice with salt, which rapidly reduced the water temperature to 2.2 °C. It took approximately 300 seconds for all test groups to approach equilibrium, with final temperatures of 2.4. °C for cubed ice in trauma bay water, 1.4 °C for cubed ice in cold tap water, 1.2 °C for crushed ice in cold tap water, and 0.2 °C for crushed ice with salt in cold tap water.

**Conclusion:**

A mixture of cold tap water, crushed ice, and sodium chloride achieved a lower equilibrium temperature and cooled more rapidly than mixtures lacking salt, using cubed ice, or prepared with warmer initial water temperature. These findings suggest that optimizing cold water immersion protocols with crushed ice, added salt, and the coolest available tap water may enhance cooling speed in simulated mixtures. Whether these differences translate into improved patient outcomes remains to be determined.

## INTRODUCTION

Heat stroke is a medical emergency that will rapidly progress to irreversible end organ damage and death if not promptly treated. The diagnosis is characterized by core body temperature > 40.5 °C and altered mental status, often with hemodynamic collapse, and a severe inflammatory response;^1^ mortality can exceed 50% within the first 28 days.^2^ With rising global temperatures, urbanization, and amphetamine epidemics the rates of heat stroke are steadily climbing. In Maricopa County, Arizona, for instance, heat-related deaths doubled between 2020 and 2023 and have increased by eight times over the previous 10 years.^3^ Given that 2024 was the hottest year recorded globally, optimizing the speed of cooling for heat stroke patients by quickly obtaining the lowest possible water bath temperature is a variable that merits attention.^4^

Once heat stroke is recognized, the core modality of treatment is rapid cooling as every minute with core body temperature sustained above 40 °C leads to increased mortality and morbidity.^5^ Expert recommendations target normothermia within 30 minutes from recognition of heat stroke symptoms.^6^ Cold water immersion is the most effective treatment, achieving cooling rates of 0.20 to 0.35 °C per minute.^7^ This method involves submerging the patient in a bath of ice and water. Although simple in concept, the logistics of implementing cold water immersion can be challenging. In an ideal scenario one can imagine a tub of ice and water premixed to a target water temperature ready for use at a moment’s notice. This setup may be available at major sporting events or military training centers where exertional heat stroke may be anticipated; however, it is not feasible in emergency departments (ED), emergency medical services vehicles, and other settings where heatstroke is less routine. In these settings it is necessary to create the optimal ice water mixture from scratch.

A 2025 paper in the *Annals of Emergency Medicine* describes a comprehensive protocol for cold water immersion that uses a body bag filled with 44 quarts of ice and 22–44 quarts of room temperature water to create a slurry.^8^ From a practical standpoint, this general guideline will suffice for implementation of the protocol in the ED setting. However, there is limited research into key variables such as the initial temperatures of the water and ice, the optimal ice-to-water ratio, the impact of ice shape (crushed, shaved, or cubed), and whether additives like salt can enhance cooling efficiency. The optimal components and technique of creating a cold-water immersion mixture remain undetermined, especially when considering real-world resource constraints.

When cold water immersion is performed the primary conduit for heat transfer from the patient is the water component, which has far greater surface area contact with the patient than the ice component. The purpose of adding ice is not to directly cool the patient but to cool the water, which in turn cools the patient. Since water rather than ice is the primary medium for heat transfer, there is an inevitable delay in cooling as the mixture reaches equilibrium. In this study we aimed to systematically evaluate key factors influencing the rapid creation of an effective ice-water mixture. Specifically, we examined the role of tap water temperature, ice temperature, ice shape, and the potential benefit of adding salt to the mixture.

**Population Health Research Capsule**

What do we already know about this issue?

*Heat stroke has high morbidity and mortality and requires rapid cooling for best outcomes, which is most effectively achieved with ice water immersion protocols*.

What was the research question?

*Is there potentially a way to improve ice water immersion protocols by adjusting ice form, water temperature, and adding salt?*

What was the major finding of the study?

*Crushed ice with salt cooled water to 0.2 °C, 2.2 °C colder than unsalted mixtures with cubed ice, reaching equilibrium faster*.

How does this improve population health?

*Improvements to cold water immersion protocols may combat rising rates of heat-related deaths in vulnerable populations*.

## METHODS

We identified several potential sources of water for cold water immersion therapy in our ED. These sources included faucets located in each of our three trauma bays, a handwashing station across from the trauma bay, and a bathroom down the hall from the trauma bay. Faucets were set to cold and allowed to run for two minutes, at which point the temperature of the water was measured and recorded. Significant discrepancies in temperature were noted between the trauma bay faucets and hallway or bathroom faucets. This observation guided the design of four cold water immersion mixtures.

We tested four mixtures of tap water and ice, with one mixture also containing sodium chloride (salt): 1) cubed ice (store bought, −1.11 °C) mixed with cold tap water from the hallway sink; 2) crushed ice (from the hospital café, −1.11 °C) with cold tap water from the hallway sink; 3) crushed ice (from the hospital cafe, −1.11 °C) with cold tap water from the hallway sink and salt; and 4) cubed ice (from the trauma bay freezer, −14.22 °C) with tap water from trauma bay 1. Each experiment was conducted with 12 quarts of ice and 12 quarts of water (11.36 liters) with four pounds of rock salt added to the crushed ice in the salt experiment. For each scenario the sourced ice originated from tap water without significant saline concentration.

At the start of each experiment we set a timer, and all components specified were poured into a 40-quart bucket. As the mixture was briefly agitated to ensure even distribution of components, we measured the initial temperature. Subsequently, the temperature of each mixture was measured at 20-second intervals for a total duration of 300 seconds (five minutes). We used a standard food-grade thermometer inserted at random locations to a depth of approximately two inches below the waterline. Data were recorded and graphed on a logarithmic axis for improved pattern visualization.

## RESULTS

Faucet water temperature varied significantly at different locations around the ED. The water source in the resuscitation/trauma bay, where most heat stroke patients are treated, was found to be 35 °C (95 °F) on the cold setting even after running for two minutes. In contrast, nearby hallway and bathroom sinks ran at 24 °C (75 °F) (Table). Room temperature during the experiment was 25.0 °C.

The data compiled from our experiments measuring the temperature of cooling mixtures (ice cubes in tap water, crushed ice in tap water, crushed ice with salt in tap water, and ice cubes in trauma bay water) revealed substantial differences in the rate of cooling and achieving depth of equilibrium temperature. After 100 seconds, water from the trauma bay with cubed ice was the warmest, reaching 6.2 °C. Tap water with cubed ice ended slightly cooler at 5.5 °C, while crushed ice in tap water reached a lower temperature of 3.6 °C. The coldest mixture was crushed ice with salt in tap water, which rapidly reduced the water temperature to 2.2 °C. By 300 seconds all test groups approached equilibrium with final temperatures of 2.4 °C for cubed ice in trauma bay water, 1.4 °C for cubed ice in tap water, 1.2 °C for crushed ice in tap water, and 0.2 °C for crushed ice with salt in tap water (Figure).

## DISCUSSION

Our experiments demonstrated that a mixture of crushed ice with salt and cold tap water can reach a lower equilibrium temperature and do so more quickly than variations of other mixtures commonly used for cold water immersion. Additionally, the water obtained from the trauma bay was warmer than expected and took longer to achieve an adequate equilibrium temperature for cold water immersion, which highlighted the importance of lower initial water temperature in cold-water immersion protocols. We did not study whether these differences would be impactful when cooling heat-stroke patients. However, it is logical and mathematically consistent to conclude that a colder cold-water immersion mixture would yield faster cooling rates in hyperthermic patients.

For example, cooling during cold water immersion approximately follows Newton’s law of cooling: *dT/dt = k(T_body_ – T_bath_)*. With a relatively small absolute change in *T_body_* the formula can be simplified to a linear relationship in which the rate of decline in core body temperature is proportional to the temperature gradient between the body and the water bath. Empirical data and reasonable derivations suggest k values between 0.005–0.013 minutes^−1^.^7^,^9^,^10^ Using a representative k = 0.013 minutes^−1^, reducing bath temperature from 2.4 °C to 0.2 °C would increase the gradient from 39.6 °C to 41.8 °C, and the predicted average cooling rate from 0.51°C/minutes to 0.54 °C/minutes, shortening cooling time by roughly 0.41 minutes for a 4 °C reduction in core temperature. These findings could have significant clinical implications and should guide additional investigation into the effectiveness of hyperthermic treatment protocols. While establishing ideal ice, water, and salt additive ratios would require high-fidelity modeling or clinical trials, several key generalizable principles can still be identified based on our experiments and fundamental physical properties.

Mathematically, the transfer of heat by convection from water to ice is proportional to the amount of surface area of contact between the two mediums. Thus, heat transfer is substantially greater with crushed or shaved ice in comparison to larger ice cubes. This effect was reflected in our experiments, with crushed ice producing a more rapid drop in water temperature than cubed ice. When possible crushed or shaved ice should be used in cold-water immersion protocols. Alternatively, if only ice cubes are available, physically breaking up the ice before use in a cold-water immersion mixture may improve cooling efficiency.

It should again be emphasized that the fluid component of cold-water immersion therapy is the primary medium for heat exchange; therefore, it stands to reason that the starting water temperature may be one of the most critical factors for a successful cold-water immersion mixture. When implementing a cold-water immersion protocol the selection of water source should be determined with the temperature of the water in mind. Our measurements of water temperature across the ED revealed that trauma bay faucets, while conveniently located, provided unacceptably warm water and could cause delays in cooling if used as the water source for cold water immersion. Alternative sources of water proved much more effective. Any medical facility seeking to implement a cold-water immersion protocol should make note of where water will be sourced and measure water temperature to ensure that the faucet temperature is in line with the goals of cold water immersion. If cold faucet water is not readily available, storing several gallons of refrigerated water at approximately 4 °C may provide a practical solution. Future studies could investigate whether ice is even necessary when the initial water temperature is sufficiently low.

Although not previously considered or studied for use in cold water immersion, the addition of salt to an ice water mixture can facilitate more rapid cooling. By disrupting intermolecular bonds in water, salt lowers the freezing point and accelerates the phase transition of ice from solid to liquid. This results in a lower latent heat of fusion and a decreased freezing point, offering two potential advantages for cold water immersion. First, converting solid ice to liquid increases surface area contact with the patient, enhancing convective cooling. Second, the liquid component of the mixture can reach lower temperatures without freezing, theoretically as low as −21.1 °C in a saturated salt solution.

From our experimentation we found that the addition of salt to an ice water mixture did produce a more rapid and profound drop in temperature. Salt is lightweight, inexpensive, easy to transport, and in theory could increase the efficiency of cold water immersion. However, prior to adopting the use of salt in cold water immersion clinically it will be important to further investigate the effect of dissolved salt on electrical monitoring equipment and defibrillation. While pure water has low electrical conductivity and previous case studies and simulations suggest defibrillation is safe in submerged patients, salt dissociates into ions that significantly increase conductivity and could increase the risk of redirecting electrical conduction pathways.^11^

## LIMITATIONS

While recording during our experiments it is likely that the temperature probe made intermittent contact with ice rather than the surrounding water mixture. This may have led to an artificially low reading of 0.1° C at 20 seconds for the crushed ice and tap water test case. A perfectly mixed ice-water slurry in which temperature equilibrates more evenly would not have this problem, but in real-world testing a probe in contact with a solid ice particle may register an exceptionally low temperature. This discrepancy was not observed in the crushed ice with salt condition where the rapid dissolution of salt facilitates uniform cooling and prevents localized cold spots. Repeating our experiment in a controlled environment could help clarify outliers and provide statistical significance to the differences we observed.

An additional limitation is the absence of clinical testing of our cooling mixtures on patients with heat stroke or volunteer subjects, which prevents direct assessment of their performance in humans. While it is reasonable to expect that colder cold-water immersion mixtures would cool hyperthermic patients more rapidly and prior studies have shown that water temperatures of 1–5 °C cool more effectively than 20–26 °C, differences in cooling rates within the 1–17. °C range have not yet been shown to be statistically significant.^12^

Future studies should evaluate the feasibility of implementing these recommendations in clinical settings and assess their impact on patient outcomes. Developing standardized guidelines based on these findings could improve the efficiency of heat stroke treatment protocols.

## CONCLUSION

The results of this study support the adoption of standardized cold-water immersion protocols that incorporate ice shape considerations and optimal water selection, ensuring more consistent and potentially effective treatment for heat stroke patients. Our study highlights several key findings that could enhance the efficiency of cold water immersion for heat stroke treatment in the emergency care setting. We found that crushed ice cools water more rapidly than ice cubes, likely due to increased surface area, and that the addition of salt further accelerates the cooling process by lowering the freezing point of water. We identified important variability between faucet temperature in the ED and, therefore, advise that medical staff monitor faucet temperatures to ensure that the water source for cold water immersion is as cold as possible. While our results suggest meaningful improvements to current practices, further research is needed to assess the clinical significance and feasibility of incorporating salt into cold water immersion, especially regarding its impact on electrical safety and medical monitoring equipment. Implementing these findings could improve the speed and effectiveness of heat stroke management, ultimately reducing morbidity and mortality associated with severe hyperthermia.

## Figures and Tables

**Figure:**
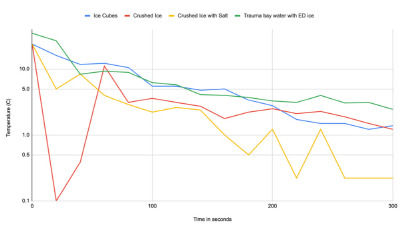
Line chart depicts the change in water temperature of four ice-water mixtures using different ice forms, water sources, and the addition of salt. Temperature measurements are shown in degrees Celsius on a logarithmic axis. *ED*, emergency department.

**Table:** Measurements of water temperature from faucets in our emergency department in a study designed to determine the quickest way to lower water temperature for treating heat-stroke patients.

	Faucet temperature set to cold after 2 minutes of running water (°C)
Trauma bay 1	35.00
Trauma bay 2	35.56
Trauma bay 3	35.00
Bathroom sink	23.89
Hallway sink	23.89
